# The Autonomous Glycosylation of Large DNA Viruses

**DOI:** 10.3390/ijms161226169

**Published:** 2015-12-09

**Authors:** Francesco Piacente, Matteo Gaglianone, Maria Elena Laugieri, Michela G. Tonetti

**Affiliations:** Department of Experimental Medicine and Center of Excellence for Biomedical Research, University of Genova, Viale Benedetto XV, 1, 16132 Genova, Italy; francesco.piacente@unige.it (F.P.); matteo.gaglianone@edu.unige.it (M.G.); melena.laugieri@gmail.com (M.E.L.)

**Keywords:** NCLDV, chloroviruses, *Phycodnaviridae*, *Mimiviridae*, glycosyltransferases, *N*-acetyl-d-glucosamine, l-fucose, l-rhamnose, d-viosamine, *N*-acetyl-l-rhamnosamine

## Abstract

Glycosylation of surface molecules is a key feature of several eukaryotic viruses, which use the host endoplasmic reticulum/Golgi apparatus to add carbohydrates to their nascent glycoproteins. In recent years, a newly discovered group of eukaryotic viruses, belonging to the Nucleo-Cytoplasmic Large DNA Virus (NCLDV) group, was shown to have several features that are typical of cellular organisms, including the presence of components of the glycosylation machinery. Starting from initial observations with the chlorovirus PBCV-1, enzymes for glycan biosynthesis have been later identified in other viruses; in particular in members of the *Mimiviridae* family. They include both the glycosyltransferases and other carbohydrate-modifying enzymes and the pathways for the biosynthesis of the rare monosaccharides that are found in the viral glycan structures. These findings, together with genome analysis of the newly-identified giant DNA viruses, indicate that the presence of glycogenes is widespread in several NCLDV families. The identification of autonomous viral glycosylation machinery leads to many questions about the origin of these pathways, the mechanisms of glycan production, and eventually their function in the viral replication cycle. The scope of this review is to highlight some of the recent results that have been obtained on the glycosylation systems of the large DNA viruses, with a special focus on the enzymes involved in nucleotide-sugar production.

## 1. Introduction

Glycosylation represents the most abundant protein post-translational modification; the addition of carbohydrates to proteins is able to significantly modify their properties, like stability, solubility, resistance to proteases, and biological activity. Moreover, glycosylation is fundamental to promote protein folding and quality control and it affects cell-to-cell interactions. All cellular organisms are able to glycosylate their proteins to some extent, using mechanisms that are generally well conserved though all domains of life. Additionally, viruses are often highly glycosylated on their surfaces and the presence of glycans affects their virulence [1]. Eukaryotic viruses studied so far use the host ER/Golgi system to produce their envelope or surface glycoproteins [1]. For instance, the presence of *N*-linked glycans, through the host ER quality control mediated by the calnexin/calreticulin system, promotes folding and trafficking of the viral glycoproteins. Moreover, the presence of sugars can be essential for interaction with host receptors and mutations causing addition and removal of glycosylation sites are often used to mask or unmask epitopes, thus contributing to immune system evasion [2]. In some cases, viruses can also affect the expression and activity of the elements of the host glycosylation machinery, in order to modify the final glycan structures [3].

In addition to hijacking and modifying the host systems, some viruses have been reported to encode their own glycosyltransferases. These enzymes are used to modify host molecules, for instance to inhibit their functions, as it is happens for baculovirus-encoded ecdysteroid glucosyltransferase. As an alternative, phage-encoded enzymes can add glucose units to their DNA during infection, to prevent the activity of host restriction enzymes. In other cases, phage glycosyltransferases induce modification in the host serotypes through lysogenic conversion, thus contributing to the evolution of pathogenic bacteria. A comprehensive review of glycosyltransferases involved in these functions can be found in [4]. However, in these cases, the glycosyltransferases are not used to modify the virus structural glycoproteins.

Recently, a novel concept for viral glycosylation has emerged, after the identification and characterization of new large DNA viruses infecting eukaryotes. These viruses, belonging to the Nucleo-Cytoplasmic Large DNA Viruses (NCLDV) group, and specifically to the *Phycodnaviridae* and *Mimiviridae* families, are characterized by a huge size compared to the conventional viruses [5,6,7,8]. Their genomes are also very large, encoding up to one thousand proteins and reaching sizes comparable to that observed for cellular organisms. Genome sequencing revealed the presence of several genes involved in glycosylation, including glycosyltransferases and other carbohydrate-modifying enzymes [9]. However, differently from the other previously-identified glycosyltransferase-coding viruses, genomes of large DNA viruses often present enzymes responsible for the production of the nucleotide-sugars that are the substrates for glycan formation. Thus, this also configures the existence of novel virus-encoded glycosylation machineries that are independent from the host for substrate supply. The scope of this review is to highlight some of the recent results that have been obtained on the glycosylation systems of the large DNA viruses.

## 2. Nucleo-Cytoplasmic Large DNA Viruses (NCLDV)

NCLDV group (also called Megavirales order) comprises several families; they infect diverse animals, algae, and protista, suggestive of an early radiation of the NCLDV, possibly concomitant with eukaryogenesis [10,11,12,13]. Their common origin from a single ancestor has been established by phylogenetic analysis of a set of essential conserved core genes; however, a strong debate is still ongoing about their origin and evolution [14]. The genomes of some members are larger and display higher complexity than the smallest cellular organisms; phylogenomic analyses have revealed that, besides the virus-specific core genes, most genes appear to be of eukaryotic origin, with a minority of them of bacterial and archaeal descent [14]. NCLDV genomes, together with typical viral proteins, encode a diverse repertoire of proteins with cell-like properties, such as components of the translation system, proteases and other elements of the protein degradation machinery, enzymes involved in redox reactions and metabolic pathways, including glycosylation.

NCLDV replicate in well-defined cytosolic regions, named virus assembly centers or “viral factories” [15,16,17,18]. For *Phycodnaviridae* and *Mimiviridae*, the typical icosahedral virions are found at the periphery of these areas. Fully-infective particles are formed in these structures, which are probably the place where glycosylation also occurs. Even if recent evidence has indicated that the inner viral membranes probably derive from ruptured host ER vesicles, there is so far no evidence indicating a direct involvement of the host machinery in viral glycan formation. Indeed, several observations, and also recent data on PBCV-1 Vp54-associated glycan structure, strongly argue against an active role of the host [19].

## 3. Phycodnaviridae

Phycodnaviruses are polyhedral viruses with a 150–190 nm diameter, containing dsDNA genomes up to 560 kb [20,21,22,23,24]. They mainly infect unicellular and pluricellular algae and are diffused in both sea and fresh waters. Increasing evidences indicate that they can affect algal population dynamics, contributing to the termination of massive algal blooms, thus representing key players in the global carbon and sulfur cycles [25,26]. The best-characterized members of the *Phycodnaviridae* family are chloroviruses. They infect unicellular chlorella-like green algae and the prototype member is PBCV-1, which was isolated in van Etten’s laboratory more than 30 years ago [27]. PBCV-1 infects *Chlorella variabilis*, which is normally found as an endosymbiont of the protozoan *Paramecium bursaria*. Since this first isolation, up to forty other chloroviruses have been identified from other chlorella hosts and their genomes were sequenced and annotated [28]. PBCV-1 and other chlorovirus properties have been extensively reviewed elsewhere [20,21,22,23].

PBCV-1 dsDNA genome contains about 330 kbp, encoding more than 400 proteins, and it has cross-linked hairpin termini. PBCV-1 major capsid protein Vp54 comprises about 40% of all virion proteins and it is coded by *a430l* gene; the protein has a predicted weight of 49 kDa, but it reaches 54 kDa due to glycosylation [29,30]. The crystal structure of PBCV-1 major capsid protein Vp54 revealed the presence of four N-linked and two *O*-linked glycosylation sites [31].

**Figure 1:**
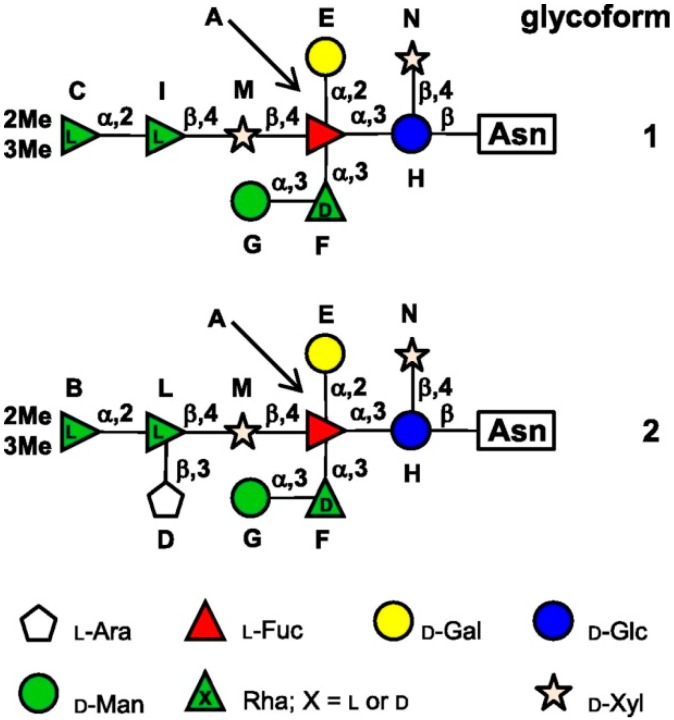
Structure of the *N*-linked glycans associated to the PBCV-1 major capsid Vp54 (from [19]). Two major glycoforms were found associated to Vp-54 protein, differing for the presence of a branching l-arabinose bound to the inner l-rhamnose.

Recently, de Castro *et al.* reported the characterization of Vp54 associated N-linked glycans by using a combined approach with NMR and MALDI-TOF mass spectrometry [19]. Two major branched glycoforms were identified, reported in Figure 1. The two glycoforms differ for the presence of an additional l-arabinose bound to the inner l-rhamnose (l-Rha) in the longest branch. The common structure is formed by nine neutral sugars and it is highly unusual for several reasons. First, the glycan is bound to asparagine (Asn) via a β-glucose (Glc). This type of linkage is very rare and it was found so far in some archea and bacteria, and a single report suggested its presence also in rat laminin [19]. A second unusual feature is the presence of a fully-substituted l-fucose (l-Fuc); this type of structure has been identified only in the phosphoglycan epitope of *Trypanosoma cruzi* gp72 [32]. One of l-Fuc substituent is the uncommon sugar d-Rha: however, its presence is consistent with the identification of a PBCV-1 encoded GDP-d-rhamnose biosynthetic pathway (see next section) [33]. The terminal l-Rha is further modified by 2-*O*-metyl and 3-*O*-metyl groups, conferring further hydrophobicity to the structure. None of the modified Asn is in the typical sequon Asn-X-(Thr/Ser) for eukaryotic and prokaryotic *N*-linked glycosylation, neither a common sequence pattern could be recognized for all glycosylation sites, posing questions about the mechanisms of site recognition by the enzyme responsible for the β-Glc linkage formation [19]. The role of the PBCV-1 glycan structure is not completely clear. It is possible that they play a role in the adhesion to the host, but no conclusive data are available on this aspect [34]. Glycans could be also beneficial for stabilization of the virion structure and protection from the environment.

Together with capsid-associated glycans, chloroviruses encode enzymes for the production of the extracellular polysaccharides hyaluronan and chitin [35,36,37]. These polysaccharides are produced early in the course of infection and accumulate as hair-like fibers on the surface of the infected cells. The role of the extracellular polysaccharides in the viral replication cycle is currently unclear, also because some chlorovirus strains lack both the enzymes for hyaluronan and chitin synthesis [20].

### 3.1. Chlorovirus Nucleotide-Sugar Biosynthetic Pathways.

#### 3.1.1. GDP-l-Fucose Production

**Figure 2:**
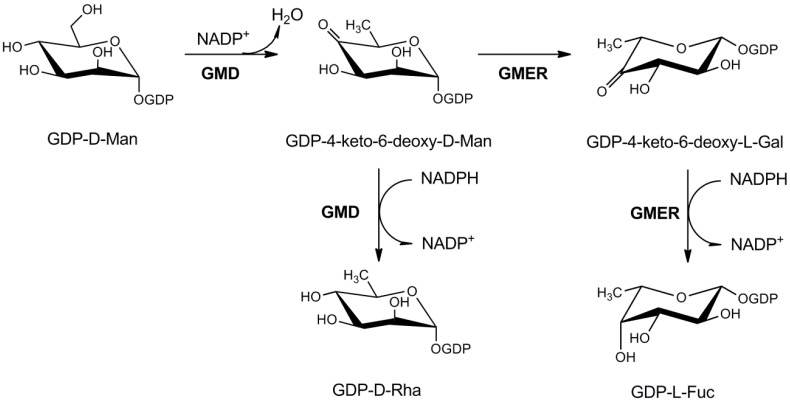
Metabolism of GDP-d-rhamnose and GDP-l-fucose in PBCV-1. PBCV-1 GMD is bifunctional, with both dehydratase and NADPH-dependent reductase activities, leading to GDP-d-rhamnose formation. GMER is a GDP-4-keto-6-deoxy-d-mannose 3,5-epimerase/4-reductase producing GDP-l-fucose.

A fully-functional GDP-l-Fuc pathway was the first nucleotide-sugar metabolism ever described in a virus [33]. PBCV-1 encodes two enzymes: a GDP-D-mannose 4,6-dehydratase (GMD, *a118r* gene), which produces the unstable intermediate GDP-4-keto-6-deoxy-mannose, and a NADPH-dependent bifunctional GDP-4-keto-6-deoxy-mannose epimerase/reductase (GMER, *a295l* gene), which catalyze the 3,5-epimerization followed by 4-reduction (Figure 2). This pathway is very well conserved though evolution and the enzymes share common properties with the bacterial and eukaryotic ones. BLAST analysis indicated that both GMD and GMER are conserved in most chloroviruses sequenced so far, indicating an essential role of this pathway in viral replication. This was quite surprising, since GDP-l-Fuc is produced by chlorella hosts; however, the cytosolic amounts of this nucleotide are usually low and GMD, the limiting step of the pathway, is subjected to strong feed-back regulation by its products. Indeed, l-Fuc is an important component of the PBCV-1 glycan core structure and, as consequence, the pathway is probably used to circumvent a limited supply of GDP-l-Fuc, which might limit the oligosaccharide synthesis.

Even if the GDP-l-Fuc pathway is conserved in all chloroviruses, the GMDs show different enzymatic properties among the different virus strains. GMD from *Acanthocystis turfacea* chlorella virus 1 (ATCV-1) is more similar to the eukaryotic ortologs, since it catalyzes the dehydration reaction, but not the reduction (Figure 2) [38]. On the other hand, PBCV-1 GMD is clearly bifunctional, since, together with the dehydratase activity, it shows also a very high NADPH-dependent reductase activity, leading to GDP-d-Rha production [33,38]. A slight GMD reductase activity was found *in vitro* also for GMDs from other species; however, the PBCV-1 GMD reductase reaction is also clearly important *in vivo*, as indicated by the presence of d-Rha in Vp54-associated glycans [19]. Preliminary data on ATCV-1 sugar composition suggest that, while l-Rha is a component of the capsid-associated glycans, d-Rha is absent, in agreement with ATCV-1 GMD catalytic activity [39]. An explanation for the different catalytic mechanism was found in the different affinities of ATCV-1 and PBCV-1 GMDs for the oxidized and reduced forms of the dinucleotide coenzyme [38]. PBCV-1 GMD has been crystallized, but the crystal resolution was not enough to explain its peculiar catalytic properties [40]. Indeed, phylogenetic analysis indicated that, while ATCV-1 GMD falls in a clade together with the bacterial enzymes, PBCV-1 GMD is not a member of a clade, indicating an extensive evolutionary divergence (Figure S1, Supplementary Materials) [38]. The reason for this difference is not clear and two hypotheses are possible: one is that PBCV-1 GMD is older than ATCV-1 GMD and that the two viruses acquired them in separate events in the course of evolution. A second more likely hypothesis is that GMD was acquired by the ATCV-1 and PBCV-1 common ancestor before their divergence; then PBCV-1 GMD evolved faster, to acquire the high reductase activity under evolutionary pressure.

#### 3.1.2. UDP-l-Rhamnose Pathway

While the enzymes for GDP-l-Fuc production are present in most chloroviruses isolated so far, the first enzyme involved in the biosynthesis of UDP-l-Rha, a UDP-d-glucose dehydratase (UGD), was found only in ATCV-1 and other few isolates, all infecting *Chlorella heliozoae*, the endosymbiont of *Acanthocystis turfacea* [41]. The second enzyme of the pathway, a bifunctional epimerase/reductase is absent. More details on the UDP-l-Rha pathway are provided below, in the section regarding the *Mimiviridae* nucleotide sugar metabolism. Sequence and phylogenetic analysis indicates that ATCV-1 UGD has been acquired by the algal host by a recent HGT (Figure S2, Supplementary Materials). Indeed, UDP-l-Rha is commonly produced by plants and l-Rha synthesis has been demonstrated to occur in Chlorella algae. Since UGD represents the limiting step for UDP-l-Rha production, it is conceivable to hypothesize that the enzyme was acquired by the virus to prevent feed-back inhibition and increase the nucleotide-sugar supply. It has to be noted that genes encoding UGD-like proteins are not present in sequenced chloroviruses infecting other hosts, suggesting that this enzymatic activity is not essential for all chlorella viruses.

#### 3.1.3. Glucosamine-6P and UDP-d-Glucuronic Biosynthesis

PBCV-1 and several other chloroviruses induce the formation of an extracellular polysaccharide, either hyaluronan or chitin, a short time after infection [35,36,37]. Two enzymes that can overcome a limited supply of the two precursors for the hyluronan biosynthesis are found in PBCV-1 genome: *a100r* gene product is a functional glutamine-fructose-6P aminotransferase (GFAT), which catalyzes the first step for the *de novo* UDP-d-*N*-acetylglucosamine (UDP-d-GlcNAc) pathway, while *a609l* gene product is a UDP-d-glucose dehydrogenase (UGDH), which promotes the NAD^+^-dependent oxidation of glucose C-6, leading to the UDP-d-glucuronate formation [42]. *a110r* is close to *a98r* gene product, the hyaluronan synthase (HAS), and in strains where *a98r* is substituted by a chitin synthase, UGDH is often lost [43]. Both GFAT and UGDH show higher homology to the bacterial enzymes, in contrast with the HAS enzyme, which is more closely related to the eukaryotic ones [42]. Indeed, a recent comprehensive phylogenetic tree of GFAT, which included also the PBCV-1 enzyme, posed this protein in a clade together with Proteobacteria, suggesting a quite recent acquisition from prokaryote organisms (Figure S3, Supplementary Materials) [44].

### 3.2. Chlorovirus Glycosyltransferases

PBCV-1 virus genome encode several proteins that display typical glycosyltransferase domains [9]. The only gene products for which the enzymatic activities have been confirmed are PBCV-1 hyaluronan synthase (HAS, *a98l* gene product) and CVK2 chitin synthase (CHS) [35,36,37]. Two chlorovirus putative glycosyltransferases have been crystallized, *a64r* gene product from PBCV-1 [45] and *b736l* from the related NY2A strain [46]. *a64r* gene product contains three distinct domains: the *N*-terminal domain shows a GTA fold similar to retaining glycosyltransferases, while the C-terminal domain resembles an *O*-methyltransferase [45]. Crystallographic studies have suggested that UDP-d-Glc is a likely substrate for the enzyme; however, the recent structural characterization of Vp54-associated glycan does not fully support this hypothesis [19]. Indeed, the *a64r* gene is restricted to few viral species and it is even absent in viruses closely related to PBCV-1 (Table S1, Supplementary Materials). Moreover, the presence of a C-terminal methyltransferase domain suggests that the enzymes may be rather responsible for the addition of the terminal l-Rha moiety and its further methylation.

The second chlorovirus glycosyltransferase for which the structure has been determined is *b736l* gene product from PBCV-NY2A virus infecting *Chlorella variabilis NC64* [46]. B736L is a type-B retaining glycosyltransferase belonging to the GT2 family; based on isothermal calorimetry, the best ligand has been identified as GDP-d-mannose [46]. However, mannose was not found as a component of PBCV-NY2A [39], suggesting that the enzyme may use another GDP-bound monosaccharide as substrate. Indeed, PBCV-1 glycans contain d-rhamnose [19], produced by GMD reductase activity on GDP-4-keto-6-deoxy-mannose [33]. *b736l* is well conserved in several species infecting *Chlorella variabilis*, including PBCV-1 (Supplemental, Table S1); sequences with low homology (about 25% identity) were also found in some species infecting *Microactinium conductrix* and *Chlorella heliozae*, such as ATCV-1 *z667l* gene product (Table S1, Supplementary Materials). However, phylogenetic analysis of these sequences has revealed that they cluster in different clades, suggesting that these PBCV and ATCV glycosyltransferases were independently acquired by the viruses [46]. It is worth noting that PBCV-NY2A and most PBCV-related species coding for sequences with high homology to *b736l* have a GMD sequence similar to the PBCV-1 type, characterized by the high reductase activity. Thus, it is conceivable that this glycosyltransferase may catalyze transfer of d-Rha to the oligosaccharide structure; however, further experiments are required to confirm this hypothesis.

Some putative PBCV-1 glycosyltransferases have orthologs with high identity in most sequenced chloroviruses, while others are confined in few species (Table S1, Supplementary Materials). Similarly, putative glycosyltransferases of the other chloroviruses are not present in PBCV-1 (not shown), collectively suggesting that chlorovirus glycosylation is complex and that differences in the final glycan structures are present. Structural studies on the glycans of representative viruses will be of great help to clarify this issue and to understand the function of the different glycosyltransferses.

## 4. Mimiviridae

The first representative of this family, *Acanthamoeba polyphaga* Mimivirus (APMV), was isolated from a cooling tower in Bradford (UK) in 1999. It was initially misidentified as an intracellular bacterium infecting *Acanthamoeba* spp., due to its large size and positivity to the Gram^+^ staining. Analysis of its morphology by electron microscopy later revealed a typical viral icosahedral morphology and its viral origin was confirmed by genome sequencing [47]. The genome comprises 1.2 Mbp, encoding more than one-thousand proteins, including several components of protein translation machinery [48]. Mimivirus capsid has a 400 nm diameter and it is covered by a dense layer of glycosylated fibers, 120–140 nm long; an inner membrane contains the nucleoprotein core. Detailed analysis of Mimivirus structure and replicative cycle have been reported elsewhere [49,50,51].

After the initial Mimivirus identification, other members of the *Mimiviridae* have been isolated, including *Cafeteria roembergensis* virus [52], *Megavirus chilensis* [53], *Moumouvirus monve* [54], and *Pheocystis globosa* virus [55], but the family is rapidly growing. All these viruses infects unicellular organism, like Amoebae and Dynoflagellatae. However, recent evidences also suggested that Mimivirus can accumulate in marine organisms used for human consumption and they may also pose hazards to human health [56,57].

Monosaccharide composition of Mimivirus fibers revealed the presence of several neutral sugars: the most abundant are Glc, GlcNAc, Rha, and the very rare aminosugar viosamine (Vio) [58]. Rha and the aminosugars are mostly associated to the fibers, while Glc was only partially depleted after fiber enzymatic removal, suggesting that Glc is bound also to other types of structures [58]. Indeed, Luther *et al.* have identified a Mimivirus glycosyltransferase, able to modify the virus collagen-like proteins with Glc units [59]. The structures of 26 different *O*-linked oligosaccharides have been obtained for Mimivirus, showing very high heterogeneity [60]. They are mainly composed by linear hexose (possibly Glc) polymers and they can be included into six structural groups: in most of them Glc and methyl-Glc are the main sugars at the reducing end, but also methyl-Vio, methyl-GlcN and methyl-Rha are found.

GC-MS analysis of *Megavirus chilensis* glycans revealed that the most abundant monosaccharide is GlcNAc, while Glc is present in low amount and Rha is absent [61]. Other sugar components are two 2-acetamido-2,6-dideoxy-l-hexoses, *N*-acetyl-l-rhamnosamine (l-RhaNAc) and possibly its epimer on C-2, *N*-acetyl-l-quinovosamine (l-QuiNAc); their 3-*O*-metyl derivatives are also present [61]. The l-enantiomers of 6-deoxy-hexosamines are very rare sugars in nature and are found in LPS and other surface polysaccharides in some Gram-positive and Gram-negative bacteria. Currently, no data are available on sugar composition for other members of the *Mimiviridae* family; however, genome sequencing has revealed the presence of putative enzymes that exhibit homologies to protein involved in the biosynthesis of other unusual nucleotide-sugars, suggesting that monosaccharide composition of *Mimiviridae* glycans can be highly variable among the different species.

### 4.1. Nucleotide-Sugar Metabolism

#### 4.1.1. UDP-d-GlcNAc Biosynthesis

The most abundant monosaccharide in Mimivirus and Megavirus outer fibers is GlcNAc. GlcNAc is found in complex glycans from all life domains and its biosynthetic pathway is present in almost all organisms studied so far. Enzymes for UDP-GlcNAc production were also found in Mimivirus [44]. The synthesis starts from fructose-6P, which is converted to glucosamine-6P using glutamine as donor for the amino group by the GFAT enzyme encoded by *L619* gene; the amino group is then acetylated by GNAT (*L316* gene) to form GlcNAc-6P. This step, where acetylation precedes the transfer of the phosphate group from C-6 to C-1, indicates that the viral pathway follows an eukaryotic-like strategy, different from the one used in Bacteria, where acetylation occurs on GlcN-1P after the mutase step [62]. No evident homolog for the eukaryotic and prokaryotic mutases, catalyzing the transfer of the phosphate group from C-6 to C-1 could be found in Mimivirus genome. Finally, the last step, the addition of a uridylyl moiety to GlcNAc-1P, is catalyzed by the *R689* gene product [44]. All the three identified enzymes were found in other members of the *Mimiviridae* family [44].

Phylogenetic analysis of the three viral enzymes revealed complex patterns (Figure S3, Supplementary Materials): while the first two enzymes are related to their eukaryotic counterparts, the last one, the uridylyltranferase, is highly homolog to the bacterial proteins. However, in all cases phylogenetic analyses pose the viral enzymes in clades separated from the cellular counterparts, suggesting that the viral pathway was acquired early in the course of evolution, possibly dating back at the beginning of giant virus evolution [44].

#### 4.1.2. UDP-l-Rhamnose Pathway

Another monosaccharide present in Mimivirus fibers in high amount is l-Rha. l-Rha is a 6-deoxyhexose and, differently from GlcNAc, it is mainly found in bacterial glycans. However, it can be also a component of complex carbohydrates in plants and fungi, for cell wall polysaccharides and for glycoside formation. In Bacteria, it is produced by the sequential activity of three enzymes that convert dTDP-d-Glc to dTDP-l-Rha [63]. The first enzyme catalyzes the removal of a water molecule from C-4 and C-6, generating an unstable intermediate, dTDP-4-keto-6-deoxy-d-Glc; this compound is then epimerized on C-3 and C-5 by an enzyme belonging to the cupin superfamily and reduced on C-4 by a NAD(P)H-dependent reductase leading to dTDP-l-Rha [63]. The dehydratase and the reductase are characterized by the presence of a typical NADB-Rossman fold. In plants the pathway is slightly different, since the initial substrate is UDP-d-Glc and the three activities are mainly found on single proteins, named RHM, which contain only two NADB-Rossman fold domains [64]. Indeed, the eukaryotic UDP-l-Rha pathway resembles the GDP-l-Fuc pathway, which does not require a separate, cupin-like enzyme for the epimerization step; an epimerase-reductase with a single NADB-domain is found [65]. Thus, in plants a UGD domain is responsible for the dehydration reaction and a second UGER domain for epimerization and reduction. Conversely, in Fungi the two domains of RMH proteins are found on separate polypeptide chains [66].

**Figure 3:**
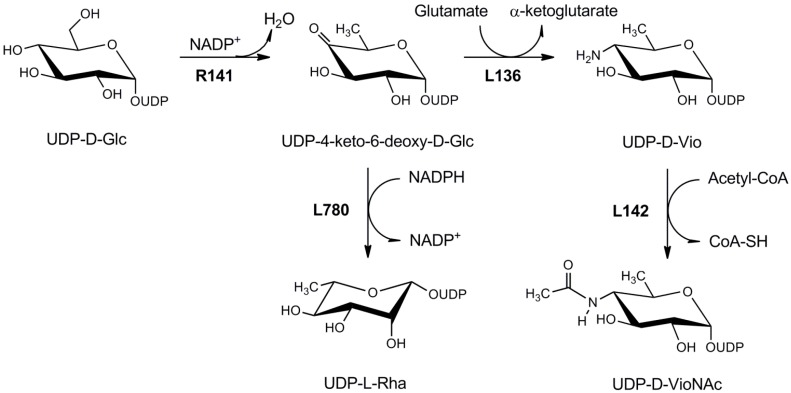
Metabolism of UDP-l-rhamnose and UDP-d-viosamine in Mimivirus. R141 is a UDP-d-glucose 4,6-dehydratase. The resulting product, UDP-4-keto-6-deoxy-glucose is the substrate for both L780, a NADPH-depdendent 3,5-epimerase/4-reductase forming UDP-l-rhamnose, and for L136, a PLP-dependent sugar aminotransferase leading to UDP-d-viosamine. The 4-amino group of viosamine is further acetylated by L142.

Mimivirus genome contains two genes coding enzymes for l-Rha biosynthesis: *R141*, which produces a UDP-d-Glc 4,6-epimerase (UGD), and *L780* for a bifunctional epimerase/reductase (UGER) (Figure 3), resembling the eukaryotic enzymes [41]. Indeed, sequence analyses revealed that viral UGD and UGER have both about 50% identity at the aminoacid level with the *Tryapanosoma cruzi* orthologs; this finding, together with analysis of gene GC content and the phylogenetic analyses, suggested that the Mimivirus did not acquire the UDP-l-Rha pathway from a recent HGT from its host, but rather it was derived from an ancient protozoan ancestor (Figure S2, Supplementary material) [41]. Thus, as proposed for the UDP-d-GlcNAc pathway, the presence of a complete UDP-l-Rha biosynthetic pathway may be used by the virus to provide sufficient amounts of the nucleotide-sugars substrate for the extensive glycan synthesis that occurs during viral replication.

#### 4.1.3. UDP-d-Viosamine Pathway

Unlike GlcNAc and l-Rha, which are produced also by eukaryotic cells, d-Vio, which is found in Mimivirus glycans, is very rare and confined to Bacteria [58]. Vio is formed by the sequential activity of UGD, the same enzyme catalyzing the dehydration step in the UDP-l-Rha pathway, and of a PLP-dependent aminotransferase that transfer an amino group from glutamine with the consequent formation of glutamate [58]. In Mimivirus, these activities are coded by *R141* and *L136* genes, respectively (Figure 3). The two genes are contained in a nine gene cluster, located at the 5′-end of Mimivirus genome. This region contains several glycogenes and, besides *R141* and *L136*, it includes glycosyltransferases and sugar-modifying enzymes [58]; the region also comprises of L142, which is a transferase catalyzing the addition of the acetyl group from acetylCoA to the 4-amino group of UDP-d-viosamine (Piacente F. *et al.*, manuscript in preparation). This cluster is lost in a Mimivirus mutant devoid of fibers, which was obtained after serial passages of the virus in axenic conditions [67].

L136 gene product shows low identity with the known bacterial enzymes that catalyze viosamine formation. The phylogenetic tree built using the *L136* sequence, reference bacterial sequences, and selected environmental sequences exhibited two main clusters: one included the bacterial sequences from different classes (cluster 2), whereas cluster 1 comprised a *L136* sequence, environmental sequences, and one of the *Shewanella sediminis* paralogous sequences of PLP-dependent transaminases, the other one belonging to cluster 2 (Figure S4, Supplementary Materials). These results suggest that the Mimivirus *L136* sequence may be ancestral and, if originating from Bacteria, it was acquired early in evolution and that it is not the results of a recent acquisition from Bacteria, which are commonly found in the *Acanthamoeba* host [58]. The finding that the first enzyme of the UDP-d-Vio pathway, the UGD, is of clear eukaryotic origin, poses further questions on how the pathway originated. In Mimivirus glycans viosamine is further modified with a 3-*O*-metyl group [58], while the 4-amino group is acetylated (Piacente F. *et al.*, manuscript in preparation).

#### 4.1.4. UDP-2-Acetamido-4,6-dideoxyhexoses

**Figure 4:**
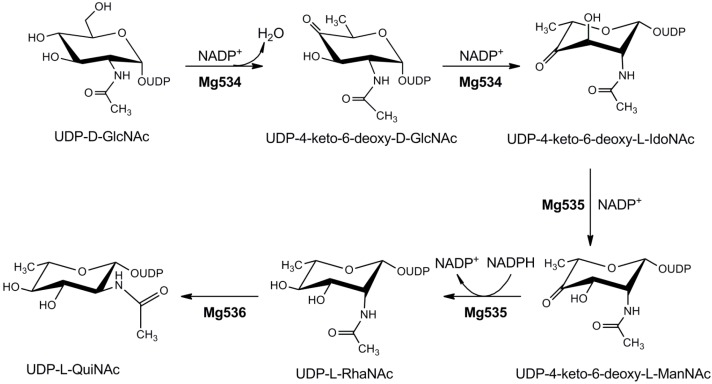
Metabolism of 2-acetamido-2,6-dideoxyhexoses in *Megavirus chilensis*. Mg534 catalyzes the 4,6-dehydration, 5-epimerization of UDP-*N*-acetyl-d-glucosamine with the formation of UDP-4-keto-6-deoxy-*N*-acetyl-l-idosamine. This compound is then epimerized on C-3 and reduced on C-4 by Mg535, to give UDP-*N*-acetyl-l-rhamnosamine. The 2-epimerase activity of Mg536, producing UDP-*N*-acetyl-l-quinovosamine has not been experimentally confirmed.

*Megavirus chilensis*, similarly to Mimivirus, encodes the UDP-d-GlcNAc pathway, but it lacks both the UDP-l-Rha and UDP-d-Vio biosynthetic enzymes. However, three enzymes of a nucleotide-sugar pathway were identified, catalyzing the formation of UDP-2-acetamido-4,6-dideoxy-l-hexoses [61]. The coding genes are localized in a cluster together with a putative glycosyltransferase containing three GT domains and other proteins involved in sugar modifications. The pathway is illustrated in Figure 4. Mg534 is an “inverting” 4,6 dehydratase, catalyzing the dehydration and 5-epimerization of UDP-GlcNAc, while Mg535 is responsible for the epimerization of C-3 and NADPH-dependent reduction of C-4, leading to UDP-l-RhaNAc. Mg536 shows high homology to bacterial enzymes that catalyze C-2 epimerization of 2-acetamido sugars; however, its enzymatic activity was not experimentally confirmed [61]. Mg534 crystallographic structure revealed the absence of the “latch”, a structure that is typically found in the bacterial homologs, suggesting that it may represent a third subfamily of the inverting UDP-GlcNAc 4,6-dehydratases (Figure S5, Supplementary Materials) [61].

### 4.2. Mimiviridae Glycosyltransferases

L230 gene product is the only glycosyltransferase of a giant virus, for which the enzymatic activity has been clearly identified; the enzyme catalyzes the transfer of a glucose unit to hydroxylysine of Mimivirus collagen-like proteins [59]. The *N*-terminal glucosyltransferase domain is associated to a C-terminal hydroxylase domain, which generates hydroxylysine. Several other putative glycosyltransferases are present in the genome of Mimivirus and other Megaviridae: however, no data have been obtained as yet on these sequences. Interestingly, some of the glycosyltransferases are clustered in genomic regions together with the enzymes involved in nucleotide-sugar metabolism [58,61]. Studies on *Mimiviridae* glycosytransferses just started; however, the identification of the glycan structures is a prerequisite to clearly identify their catalytic function.

## 5. Conclusions

Evidence accumulated in the past years indicate that chloroviruses, and possibly Mimivirus and *Megavirus chilensis*, own autonomous glycosylation systems, which are independent for both the supply of the monosaccharide substrates and the glycosyltransferases required to build up the glycan structures. However, genome analyses indicate the presence of putative glycogenes also in other members of the *Phycodnaviridae* and *Mimiviridae* families and in some newly discovered viruses of the NCLDV group. Prasinoviruses (*Phycodnaviridae*) infecting *Ostreoccus*, *Bathycoccus*, and *Micromonas* Prasinophyceae species present clusters encoding for several genes for nucleotide-sugar metabolism and glycosyltransferases, suggesting the presence of complex glycosylation pathways [68]. Similar results are obtained also for *Mimiviridae*, such as *Pheocystis globosa* virus [55] and *Cafeteria roembergensis* virus [52]. Some putative glycosyltransferases are found also in the genomes of the *Pandoraviruses* [69], *Pithovirus sibericus* [70], and *Mollivirus sibericus* [71], suggesting that the presence of virus-encoded glycosylation systems may be more widespread than previously thought.

However, these studies are still at the beginning and many issues need to be addressed. First, accumulating evidence indicates that the NCLDV have a long evolutionary history, possibly dating from the divergence of Eukaryotes and Prokaryotes [14], so that comprehensive study of the glyco-enzymes may contribute to understand the origin and evolution of today’s cellular glycosylation pathways. Indeed, it is not clear how the autonomous glycosylation pathways of Chloroviruses and *Mimiviridae* originated. Phylogenetic analyses of the enzymes indicate complex patterns of acquisition, from both prokaryotes and eukaryotes, even in the same pathway, as observed for the UDP-d-Vio synthesis in Mimivirus [58]. In other cases, enzymes are highly divergent from the cellular homologs, suggesting either a very early acquisition from a host by a viral ancestor or an accelerated evolution of more recently acquired viral genes. Studies on other viruses will help to clarify this issue.

Another very important aspect that needs to be investigated is how the glycosylation process is organized inside the virus factories. Several evidences have shed light on the formation of the cytosolic areas where virus assembly occurs [15,16,17,18], but nothing is presently known on how carbohydrate addiction occurs. Moreover, the role of glycosylation on virus replicative cycle and infectivity is not completely clear and needs to be explored. The data obtained so far also highlight the need to use rare sugars, which are not produced by the eukaryotic hosts, to obtain unusual oligosaccharide structures. These structures are probably essential for the interaction of the viral particles with the host, in particular to promote phagocytosis, but they can also provide protection against the harsh environment in which the viruses need to propagate. Indeed, glycans, in particular those mimicking the surface of Bacteria, are important to trigger host phagocytosis response, in particularly for viruses that use this mechanisms for infection, as *Mimiviridae*, but they can also provide protection and promote survival in the host phagosomes. It is worth noting that also a chlorovirus, ATCV-1, which uses a phage-like strategy for infection, can persist in mammalian macrophages, simulating an inflammatory response, which has been related to a decrease in cognitive functions [72,73]. The impact of large DNA viruses on human health is still highly controversial; however, besides effects due to virus replication, exposure to viral glycans could also affect the immune response and even it might contribute to the development of autoimmunity, as it occurs for bacterial glycoconjugates.

Finally, identification of virus encoded glycogenes could also be seen in a biotechnological perspective, resulting in practical glycotechnological applications. They would expand the repertoire of enzymes already used in platforms for producing glycoconjugates and for modification of proteins. Since the glycosyltransferases lack a predicted transmembrane domain, the recombinant proteins are expected to be soluble, thus making their production easier. Moreover, the recombinant viral enzymes for nucleotide-sugar production are often more stable and with different properties compared to the cellular homologs; thus, they could represent useful tools for the production of rare sugars.

## Supplementary Materials

Supplementary materials can be found at http://www.mdpi.com/1422-0067/16/12/26169/s1.
