# Supplementary material for: The Autonomous Glycosylation of Large DNA Viruses
Source: Int J Mol Sci. 2015 Dec 9;16(12):29315–28. doi: 10.3390/ijms161226169 (PMC4691112; doi:10.3390/ijms161226169)
Supplement: Supplementary file 1 [file ijms-16-26169-s001.pdf]

Supplementary Materials: The Autonomous Glycosylation of Large DNA Viruses

Francesco Piacente, Matteo Gaglianone, Maria Elena Laugieri and Michela G. Tonetti

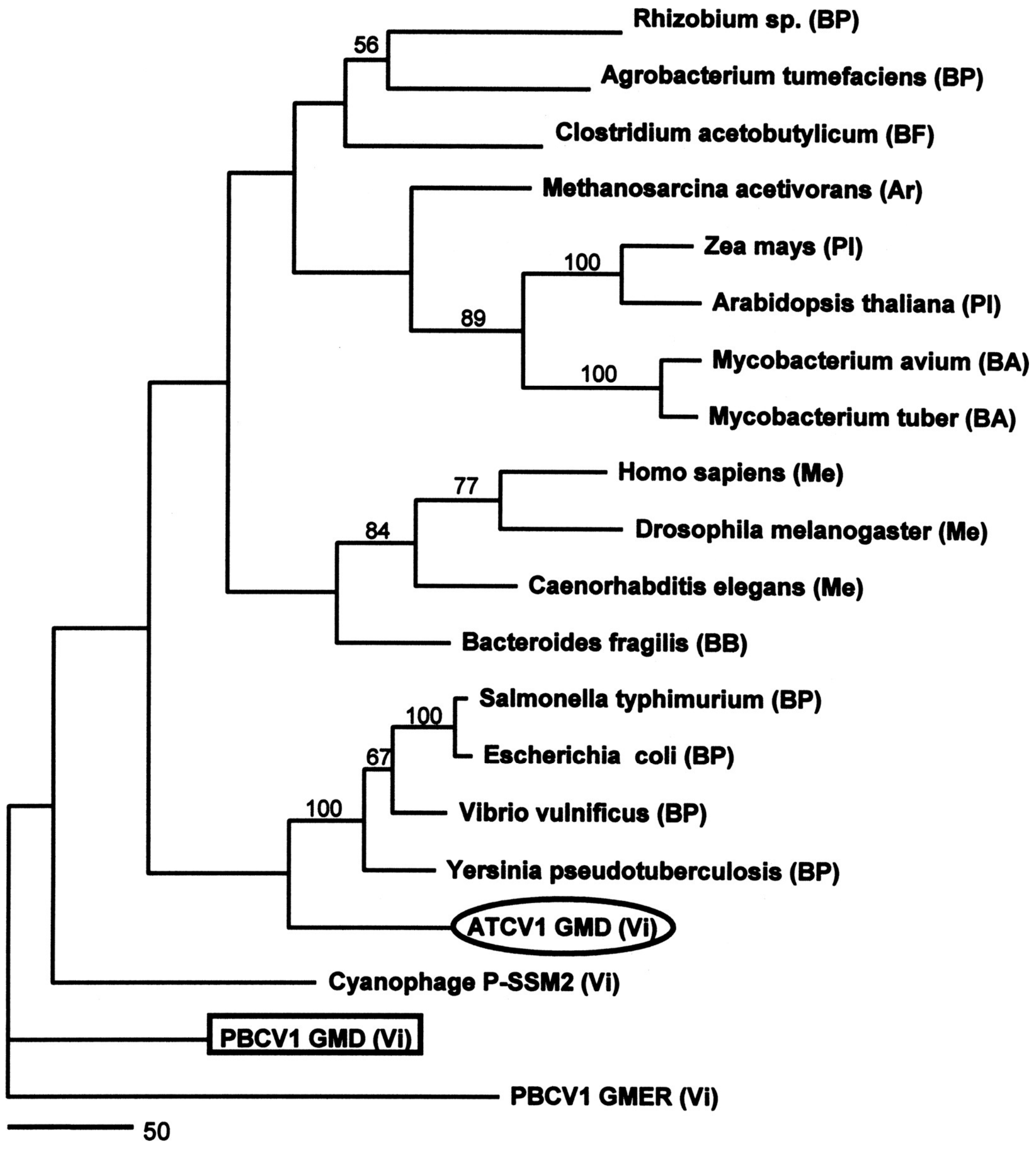

Figure S1. Rooted phylogram of GMD. A BLAST search was conducted using the virus PBCV-1 GMD protein (PBCV-1: AAC96486) [38]. This research was originally published in Journal of Biological Chemistry. Fruscione, F.; Sturla, L.; Duncan, G.; van Etten, J. L.; Valbuzzi, P.; de Flora, A.; di Zanni, E.; Tonetti, M. Differential role of NADP+ and NADPH in the activity and structure of GDP-D-mannose 4,6-Dehydratase from two chlorella viruses. J Biol Chem. 2008, 283, 184–193. © the American Society for Biochemistry and Molecular Biology.

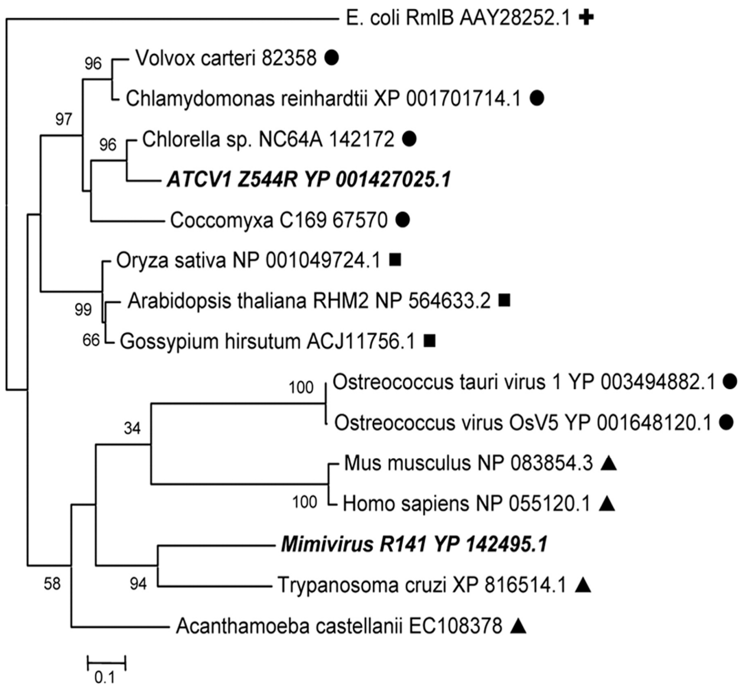

(**a**)

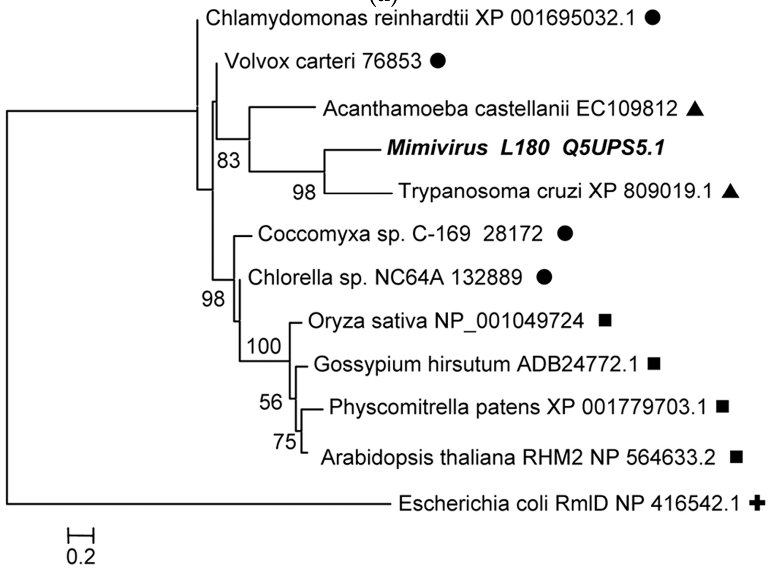

(**b**)

Figure S2. (**a**) Maximum-likelihood tree of UDP-D-glucose 4,6-dehydratase (UGD) for 14 taxa, including the viruses ATCV-1 and Mimivirus; (**b**) Maximum-likelihood tree of UDP-4-keto-6-deoxy-D-glucose epimerase/reductase (UGER) for 12 taxa, including mimivirus [40]. Parakkottil Chothi, M.; Duncan, G. A.; Armirotti, A.; Abergel, C.; Gurnon, J. R.; van Etten, J. L.; Bernardi, C.; Damonte, G.; Tonetti, M. Identification of an L-rhamnose synthetic pathway in two nucleocytoplasmic large DNA viruses. J Virol. 2010, 84, 8829–8838. Copyright © American Society for Microbiology.

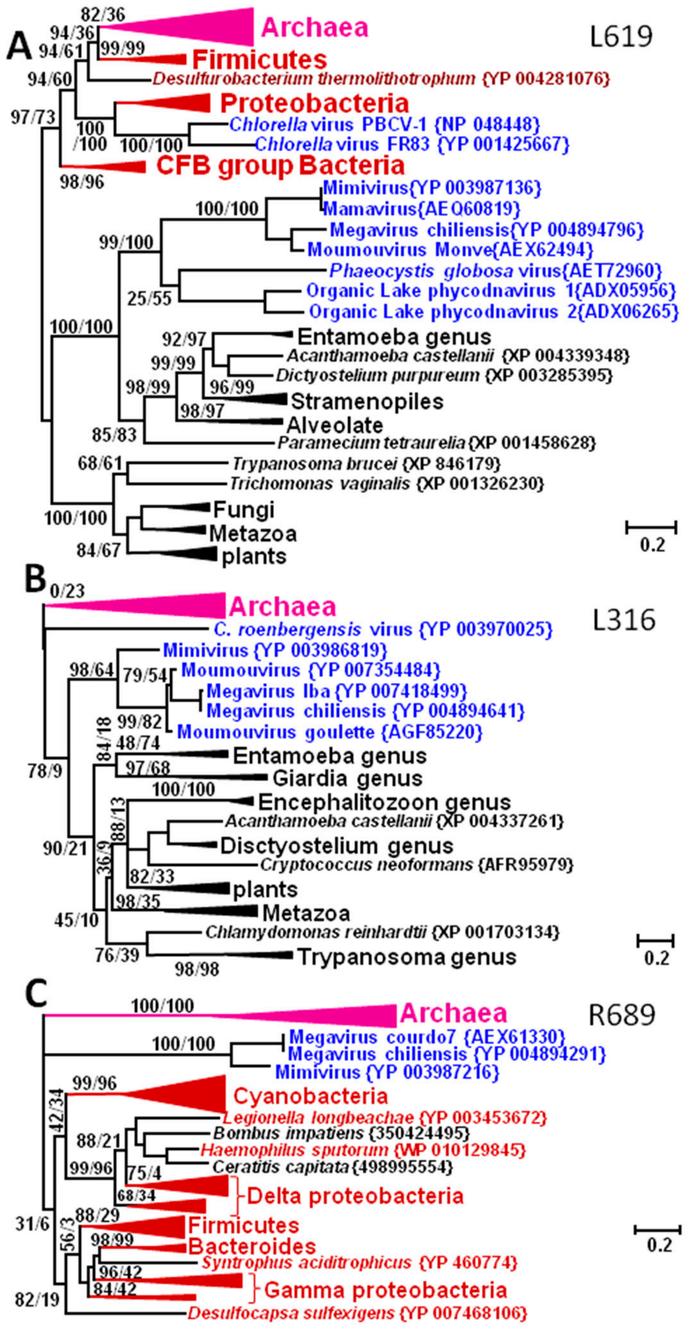

Figure S3. Unrooted phylogenetic trees of the enzymes of the Mimivirus UDP-GlcNAc pathway. (**A**) Maximum likelihood (ML) tree constructed from the multiple-alignment of GFATs containing 460 gap-free sites; (**B**) ML tree constructed from the multiple-alignment of GNATs containing 123 gap-free sites; (**C**) ML tree constructed from the multiple-alignment of UAPs containing 192 gap-free sites. The L619 tree contains also GFAT from Chlorella viruses [43]. Piacente, F.; Bernardi, C.; Marin, M.; Blanc, G.; Abergel, C.; Tonetti, M. G. Characterization of a UDP-N-acetylglucosamine biosynthetic pathway encoded by the giant DNA virus Mimivirus. Glycobiology 2014, 24, 51–61. By permission of Oxford University Press.

Table S1. Presence of PBCV-1 glycosyltransferase orthologs in other chloroviruses.

Protein Name	Family	*Chlorella Variabilis* sp. NC64A										
		PBCV-1	PBCV-AN69C	PBCV-KS1B	PBCV-NE-JV-4	PBCV-CviKI	PBCV-IL-5-2s1	PBCV-AR158	PBCV-NY-2A	PBCV-NY-2B	PBCV-CvsA1	PBCV-MA-1D
A64R	GTnc	Id>90	Id>90	Id>90	Id>90							
A98R	GT2	Id>90				Id>90	Id>90					
A111/114R	GT2	Id>90	Id>90	Id>90	Id>90	Id>90	Id>90	Id>90	Id>90	Id>90	Id>90	Id>90
A222R	GT2	Id>90	Id>90	Id>90		Id>90	Id>90	Id>90		Id>90	Id>90	
A473L	GT2	Id>90	id<50	id<50	id<50	Id>90				id<50		
A546L	GT4	Id>90	Id>90	Id>90	Id>90	Id>90	id>70	id>70	id>70			

Protein Name	Family	*Micractinium Conductrix*												
		PBCV-MT325	PBCV-Fr483	PBCV-NW665.2	PBCV-CZ-2	PBCV-OR0704.2.2	PBCV-CVG-1	PBCV-Can18-4	PBCV-CVB-1	PBCV-NE-JV-1	PBCV-Fr-5-L	PBCV-CVA-1	PBCV-CVM-1	PBCV-AP110A
A64R	GTnc													
A98R	GT2	id>70	id>70	id>70	id>70	id>70	id>70							
A111/114R	GT2	id>50	id>50	id>50	id>50	id>50	id>50	id>50	id>50	id>50	id>50	id>50	id>50	id>50
A222R	GT2	id>70	id>50	id>70	id>70		id>50	id>70	id>70	id>70	id>70			
A473L	GT2	id>70	id>70					id>70	id>70			id>70	id>70	id>70
A546L	GT4				id<30	id<30								

Protein Name	Family	*Chlorella Heliozoae* SAG 3.83												
		ATCV-1	ATCV-GM0701.1	ATCV-Can0610SP	ATCV-Br0604L	ATCV-TN603.4.2	ATCV-MN0810.1	ATCV-NTS-1	ATCV-NE-JV-2	ATCV-OR0704.3	ATCV-MO0605SPH	ATCV-NE-JV-3	ATCV-Canal1	ATCV-WI0606
A64R	GTnc													
A98R	GT2													
A111/114R	GT2	id>50	id>50	id>50	id>50	id>50	id>50	id>50	id>50	id>50	id>50	id>50	id>50	id>50
A222R	GT2	id>50	id>50	id>50	id>50	id>50			id>50	id>50	id>50	id>50	id>50	id>50
A473L	GT2	id>70	id>70	id>70	id>70	id>70	id>70	id<50	id<50	id<50	id<50	id<50	id<50	
A546L	GT4	id<30	id<30	id<30					id<30	id<30	id<30	id<30		

Legend:
- Id>90
- id>70
- id>50
- id<50
- id<30
- Not found

Colored boxes indicate the percentage of amino acid identity. Chloroviruses are divided depending on host specificity.

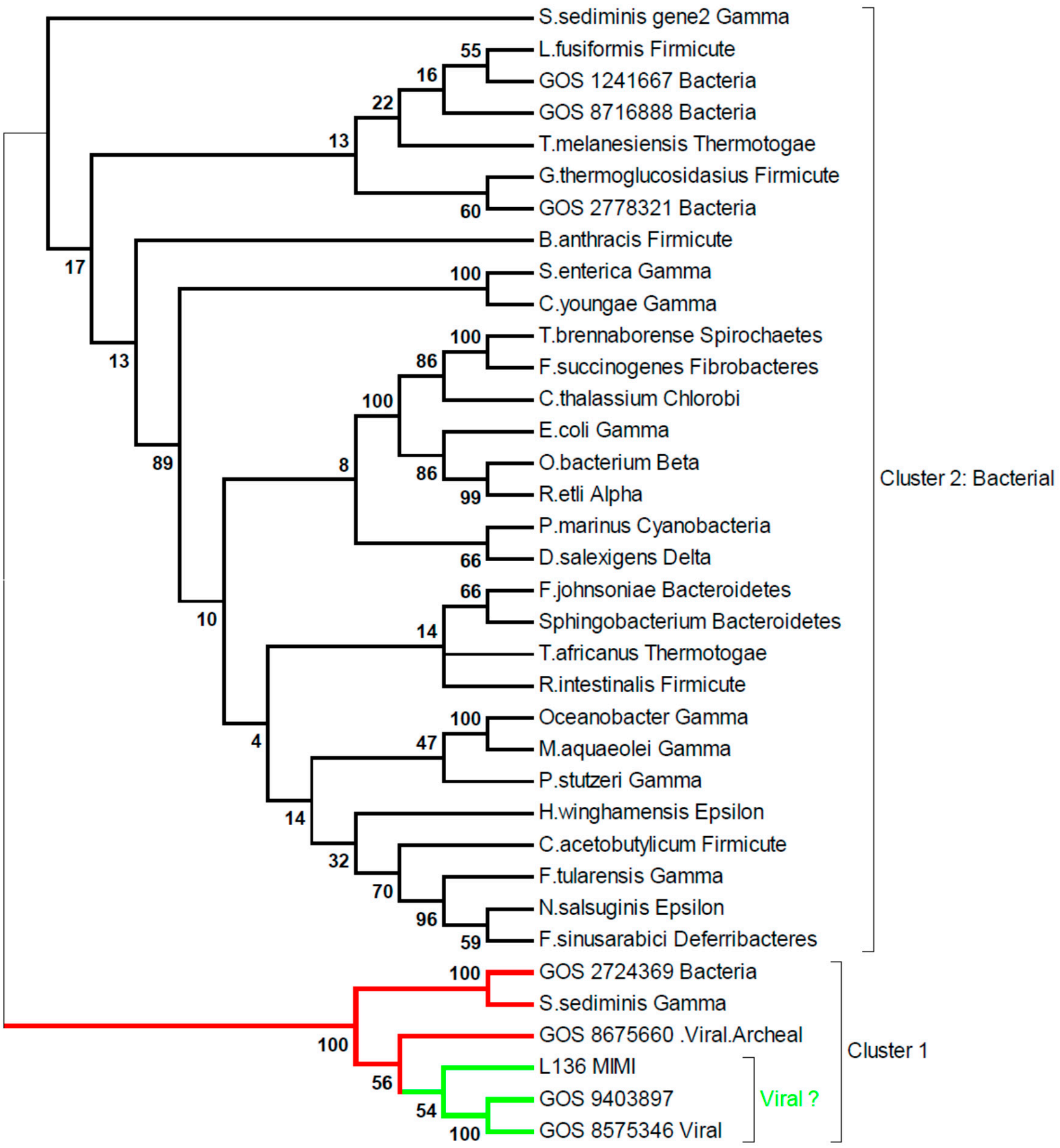

Figure S4. Unrooted phylogenetic tree of Mimivirus L136 invovled in UDP-D-Viosamine production [57]. This research was originally published in Journal of Biological Chemistry. Piacente, F.; Marin, M.; Molinaro, A.; de Castro, C.; Seltzer, V.; Salis, A.; Damonte, G.; Bernardi, C.; Claverie, J. M.; Abergel, C.; Tonetti, M. Giant DNA virus mimivirus encodes pathway for biosynthesis of unusual sugar 4-amino-4,6-dideoxy-D-glucose (Viosamine). J Biol Chem. 2012, 287, 3009–3018. © the American Society for Biochemistry and Molecular Biology.

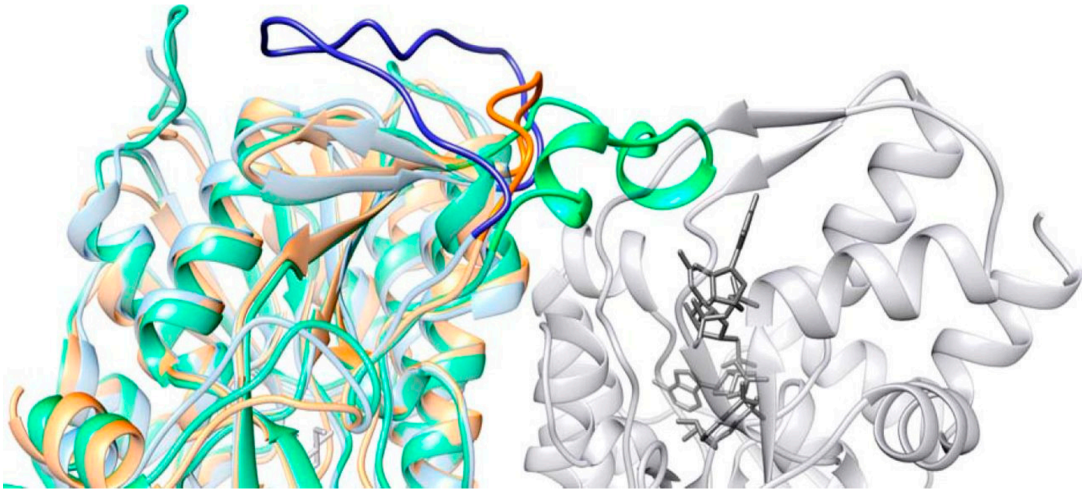

Figure S5. Ribbon representation of the "latch" region in Mg534 structure. Ribbon representation of the structure of Mg534 (PDB 4TQC) (orange) superimposed with CapE (PDB 4G5H) (green) and FlaA1 (PDB 2GN4) (blue). The view is centered on the variable region corresponding to the CapE latch. The molecule in light grey corresponds to the second monomer of the dimer of CapE. The substrate and the coenzyme are colored in dark grey. While the latch of CapE (dark green) associates with the substrate binding domain of the second monomer, the equivalent region of FlaA1 folds back on the first monomer (dark blue). This long loop is absent from the Mg534 structure [60]. This research was originally published in Journal of Biological Chemistry. Piacente, F.; de Castro, C.; Jeudy, S.; Molinaro, A.; Salis, A.; Damonte, G.; Bernardi, C.; Abergel, C.; Tonetti, M. G. Giant virus megavirus chilensis encodes the biosynthetic pathway for uncommon acetamido sugars. J Biol Chem. 2014, 289, 24428–24439. © the American Society for Biochemistry and Molecular Biology.
